# To Remember or to Forget: The Role of Good and Bad Memories in Adoptive T Cell Therapy for Tumors

**DOI:** 10.3389/fimmu.2020.01915

**Published:** 2020-08-27

**Authors:** Anna Mondino, Teresa Manzo

**Affiliations:** ^1^Division of Immunology, Transplantation and Infectious Diseases, IRCCS San Raffaele Scientific Institute, Milan, Italy; ^2^Department of Experimental Oncology, IRCCS European Institute of Oncology, Milan, Italy

**Keywords:** T cell, memory, adoptive T cell immunotherapy of cancer, competition, tumor immunity

## Abstract

The generation of immunological memory is a hallmark of adaptive immunity by which the immune system “remembers” a previous encounter with an antigen expressed by pathogens, tumors, or normal tissues; and, upon secondary encounters, mounts faster and more effective recall responses. The establishment of T cell memory is influenced by both cell-intrinsic and cell-extrinsic factors, including genetic, epigenetic and environmental triggers. Our current knowledge of the mechanisms involved in memory T cell differentiation has instructed new opportunities to engineer T cells with enhanced anti-tumor activity. The development of adoptive T cell therapy has emerged as a powerful approach to cure a subset of patients with advanced cancers. Efficacy of this approach often requires long-term persistence of transferred T cell products, which can vary according to their origin and manufacturing conditions. Host preconditioning and post-transfer supporting strategies have shown to promote their engraftment and survival by limiting the competition with a hostile tumor microenvironment and between pre-existing immune cell subsets. Although in the general view pre-existing memory can confer a selective advantage to adoptive T cell therapy, here we propose that also “bad memories”—in the form of antigen-experienced T cell subsets—co-evolve with consequences on newly transferred lymphocytes. In this review, we will first provide an overview of selected features of memory T cell subsets and, then, discuss their putative implications for adoptive T cell therapy.

## T Cell Memory and Adoptive T Cell Therapy

T cells play a crucial role in immunity against pathogens and cancer. Antigen (Ag) encounter, costimulatory signals, and pro-inflammatory cytokines dictate naïve T cell (T_N_) activation in secondary lymphoid organs, which is followed by clonal expansion and differentiation of effector T cells (T_EFF_) (1–3). Although the vast majority of T_EFF_ cells die via apoptosis after antigen clearance, stable populations of memory T cells (T_M_) can persist over time and ensure a rapid recall response upon further encounter with cognate Ag. The T_M_ pool is composed of several subsets harboring a considerable heterogeneity in trafficking, localization, effector functions, and durability; all features with direct consequences on recall responses.

The growing understanding of the cellular and molecular events underlying their behaviors, functionality, and persistence has instructed the development of defined T cell manufacturing protocols suitable for adoptive T cell therapy (ACT) against cancer and helped predict their behavior and efficacy *in vivo*. In the context of ACT, tumor-specific T_M_ lymphocytes are generally produced *in vitro* by the expansion of tumor-infiltrating T cells (TILs) derived from tumor specimens or peripheral blood, or by the genetic engineering of peripheral blood mature T cells with tumor-specific T cell receptor (TCR) or chimeric antigen receptor (CAR). The adoption of ACT envisages several steps: (1) generation of T cell products, (2) conditioning of the host, (3) T-cell transfer, and (4) post-transfer cell support. Each of these steps can have a critical impact on ACT therapeutic efficacy, and vary according to infused T cells’ T_M_ features, and simultaneously shape the immune landscape of the host. Indeed, mounting evidences indicate that the differentiation status of the transferred T cells along with tumor-intrinsic and tumor-extrinsic factors are important determinants of ACT clinical outcome (4). Once (re)infused in patients, tumor-specific T lymphocytes face the challenge to react to tumor lesions, which might vary in anatomical distribution and complexity, in the presence of a plethora of pre-existing T_M_ subsets, which might promote or oppose infused T cell activity. Although the density of CD3^+^ TILs is generally a favorable prognostic factor for responses to therapy and overall survival of cancer patients, TILs can prove hyporesponsive or exhausted, and as such represent a barrier for ACT. Here, we review some of the seminal characteristics of memory/exhausted T cell subsets [reviewed in details elsewhere (3, 5, 6)] to highlight how pre-existing T_M_ might assist or outcompete newly transferred T cells, and by that represent an advantage or disadvantage for current ACT.

## Memory T Cells Come in Different Flavors

Although T_EFF_ cells mostly disappear upon pathogen/antigen clearance, T_M_ cells survive and patrol against secondary infection or metastatic recurrence in the case of tumors (7, 8). T_M_ cells consist of a collection of distinct subsets of cells with considerable heterogeneity in phenotype, function, location, and trafficking (9, 10). Based on distinctive migratory and effector properties, circulating memory CD4 T cells were initially classified in central memory T cells (T_CM_ cells) and effector memory T cells (T_EM_ cells) (11). CD4 T_CM_ cells, similar to T_N_ cells, express the lymph node and T cell zone homing receptors CD62L and CCR7 and produce substantial amount of IL-2, but lower levels of effector cytokines and cytotoxic molecules (11). A similar phenotype also characterized memory CD8 T cells. CD4 and CD8 T_CM_ cells have good proliferative capacity in response to Ag and ability to self-renew in response to IL-7 and IL-15. Within the long-lived memory subsets, also stem cell memory T cells (T_SCM_) can be identified for their more naïve-phenotypic qualities and stem cell–like properties including the capacity to reconstitute the entire spectrum of memory and effector T cell subsets (12–15). The long-lived properties of both T_CM_ and T_SCM_ have been considered for effective vaccine design, and exploited in the setting of ACT, where they are associated with improved anti-tumor responses and therapeutic benefit. T_EM_ cells, instead, generally lack CD62L and CCR7, produce effector cytokines, and have higher cytotoxicity when compared with T_CM_. Although T_CM_ circulate between secondary lymphoid organs and blood, T_EM_ circulate between blood and non-lymphoid tissues, where they persist long after Ag clearance (16, 17). The surface expression of the chemokine receptor CX3CR1 further refines T_CM_ (CX3CR1^–^) and T_EFF_ (CX3CR1^+^), (18) and identifies an additional peripheral memory T cell subset (T_PM_ CX3CR1^int^), which appear to possess the highest steady-state self-renewal capacity of all T_M_ subsets, being able to survey peripheral tissues and return to secondary lymphoid organs, via the lymphatic system. A further distinct subset of T_M_ is constituted by tissue resident memory T cells (T_RM_ cells), which represent the front-line defense in case of reinfection, especially at barrier sites, such as the skin, lung, and gut, having rapid proliferation potential and immediate effector function capacity, (19–21) critically important in cancer immunology (22, 23). These have been described among CD4 and CD8 T cell subsets as being able to remain positioned within non-lymphoid tissues after Ag clearance and lack recirculation capacities (16, 17, 24). CD8 T_RM_ were initially characterized the by expression of CD103 [αE(CD103)β7], CD49a (VLA-1 or α1β1), and the C-type lectin CD69, critical for their retention into tissues, (25, 26) and for recruitment within epithelial tumor regions (27). More recently, data have shown that some CD8 T_RM_ cells lack CD103, and that this integrin is not an absolute marker for residency of CD4 T_RM_, which also appear more heterogeneous compared with CD8 T_RM_ (28–30). Although the origin of CD4 T_RM_ cells remains debated, recent evidences suggest they might originate from CD4 T follicular helper subsets, which share with CD8 T_RM_ some key features related to their migration, differentiation, and maintenance [reviewed in (31)]. Lastly, it is worth mentioning that also Ag-inexperienced T cells with CD44^hi^CD122^+^ memory features have been described both in mice and humans (32, 33). These can arise in the thymus (innate-like memory cells), or in response to lymphopenia (virtual-memory T cell), and can contribute to protective anti-tumor immunity (32–35).

Overall, the heterogeneity of T_M_ subsets, with defined phenotypes, functions, and anatomical distribution, contributes to effective protective immunity. This has to be taken into consideration when developing ACT-based strategies, as adoptively transferred T cell products should closely mimic the behaviors of naturally occurring cells and be endowed with both effector functions, to promote acute tumor debulking, and long-term persistence, to promote surveillance against recurrent disease.

## The Making of T Cell Memory: A Three-Signal Business

The strength and duration of TCR engagement by cognate peptide–MHC complexes (signal 1), co-stimulation (signal 2), and inflammatory cytokines (signal 3) contribute to naïve T cell priming and T_M_ differentiation (36–38). CD4 T_N_ cells require persistent TCR-peptide/MHCII interactions to achieve maximal clonal expansion (39). This can be regulated by the strength of TCR-peptide/MHCII binding (40) or by repeated contacts with Ag-bearing APCs, (41) and have direct consequences on T_EFF_ cell function (42). In contrast, CD8 T_N_ cells can be “programmed” by short-term access to Ag to allow T_EFF_ differentiation, and prolonged and stable interaction with Ag-bearing APC appear necessary for full T cell activation and memory generation (43). Costimulatory ligand/receptor pairs, able to control the magnitude of the T cell response and the rate of T_M_ development and maintenance, generally provide signal 2. CD28 and members of the tumor necrosis factor receptor (TNFR) family, such as CD27, 4-1BB, and OX-40, in particular impact the formation and/or responsiveness of the memory CD4 and CD8 T cell pool [reviewed in (44)]. The expression of costimulatory receptor can be constitutive, as in the case of CD28 or CD27, or also inducible, as in the case of OX40 or 4-1BB, in response to IL-7 and IL-15, which promote T_M_ survival and homeostatic turnover (44). The importance of costimulation in adopting the appropriate T_M_ feature has also been demonstrated in ACT (45). For instance, exogenous agonistic anti-4-1BB IgG4 significantly promoted the yield of TIL expansion, and programmed them for enhanced survival and effector functions (36). Similar CAR-T cell engineering has evolved to include costimulatory domains in the original CAR construct. Initial studies demonstrated the beneficial effect of including the intracellular domain of CD28 to elicit both TCR and CD28 activation. Later, it became clear that a range of other costimulatory domains, including ICOS, and the TNFR superfamily members 4−1BB, OX40, and CD27, could rather promote long-lived memory cells (46). In addition, the engineering of defined CD28- or 41BB-costimulatory moieties within CAR constructs was proven to favor glycolysis (CD28) or mitochondrial biogenesis and oxidative metabolism (41BB) with direct implication for memory development *in vivo* (47).

Inflammatory cytokines (signal 3) also contribute to T cell priming. They do so by promoting T cell proliferation, effector functions acquisition, and long-term maintenance of protective immunity. At least three candidate cytokines, IL-12, type-I IFNs, and IFN-γ, have been shown to differentially contribute to CD8 T_M_ cell differentiation (7, 48). In the case of CD4 T cells, according to the type of immunological insult, the host-pathogen interaction, and resulting pro-inflammatory cytokine expression, promote differentiation of various T helper subsets (T_H_1, T_H_2, T_H_17, T follicular helper cells/T_FH_, T regulatory cells/reg) with pleiotropic effector functions, some degree of plasticity, and various memory-forming potential (31, 49, 50).

Overall, the characterization of the signals dictating T cell differentiation has been instrumental to identify specific manufacturing conditions for ACT. The use of TCR and costimulatory receptor engaging ligands, in combination with common γ chain (γc) cytokines and defined nutrients, can instruct T cells to adopt different T_M_ features, with direct consequences on T cell engraftment and long-term survival.

## Not All Memories Are Good Ones: The Case of Chronic Infection and Cancer

Any interferences in the three-signal model of T cell activation can result in dysfunctional phenotypes, sometimes endowed with inhibitory functions (51). For instance, defective co-stimulation, negative co-stimulation by inhibitory receptors, or anti-inflammatory cytokines can make T cells anergic and tolerant to cognate Ag, (52, 53) or induce the differentiation of T_reg_ cells. Ag-experienced tolerant T cells, compared with T_M_, express high levels of co-inhibitory receptors (e.g., PD1, CTLA-4, TIM3, and LAG3) and transcriptional repressor (e.g., EGR1/2, DUSP2), low levels of cytokines and chemokine receptors, and mostly lack effector functions (53). In addition, continuous stimulation through the TCR, typically induced by Ag persistence during chronic infections or cancer, drives CD4 and CD8 T_M_ into a state referred to as T cell exhaustion (T_EX_ cells) (54, 55). T_EX_ cells are characterized by progressive loss of effector functions, metabolic deregulation, poor memory recall, and homeostatic self-renewal (54, 56). They acquire high and sustained expression of different inhibitory receptors, which are not found on T_M_ cells arising after resolution of an acute infection (57). Although CD8 T cell exhaustion was first described in LCMV chronic infection, (58, 59) it is now clear that it occurs in several other chronic infections, (60–62) in autoimmune disorders (63, 64) as well as in cancer (56, 65). T_EX_ cells, along with classical anergic T cells and T_reg_ emerging from the thymus or generated by the conversion of T_EFF_ cells, (66) might induce a dysfunctional state in tumor-infiltrating CTLs (67) and represent barriers to engraftment and function of ACT products. Accordingly, depleting strategies have improved responses to immunotherapy (68).

Although initially described in the context of infectious diseases, tumor-associated T_RM_ should also be carefully considered as their role in oncology has now been established (22, 69). In general terms, T_RM_ produce effector molecules such as IFN-γ, TNF-α, and IL-2 more rapidly than other memory T cells, (70) orchestrating the recruitment of auxiliary immune cells to the infected site (71). In tumor immunity, T_RM_ have nevertheless been reported to play controversial functions. For instance, CD8 T_RM_ cells driven by autoimmune vitiligo or primed by active vaccination conferred protection against melanomas, and their intra-tumoral representation was correlated with favorable prognosis in a variety of cancers (72, 73). In other studies, instead, tumor-associated CD103^+^ CD8 T_RM_ showed regulatory properties (CTLA-4 and IL-10 expression), and were found to adopt a dysfunctional phenotype over time by expressing the highest levels of PD-1, TIM-3, CTLA-4, and LAG-3 (74). It should, however, be noted that tumor-associated T_RM_ can respond to anti-PD-1 treatment and were described to exert potent cytotoxic and effector functions in melanoma patients (75). Likewise, tumor-associated CD103^+^CD4 T_RM_ have been described to be highly enriched for tumor-specific T cells (76) and suppress tumor growth through the secretion of TNF-α and IFN-γ or direct killing of tumor cells (77, 78). Again, these cells express high levels of co-inhibitory receptors, and yet whenever activated in appropriate conditions (such as by providing agonistic stimulation of CD27 or CD28 co-stimulatory molecules and/or immune checkpoint blockers) (79) might represent valuable allies and support tumor rejection. Otherwise, T_RM_ cells may be an obstacle to ACT products, as they might compete for local resources. For instance, T_RM_ cells are highly sensitive to IL-15, a cytokine which newly transferred T cells depend on (80). Thus, a better understanding of the molecular mechanisms mediating CD4 and CD8 T_RM_ differentiation and interplay will allow harnessing the protective capacity of these memory subsets and modulate their activity in the context of ACT. In this respect, tumor-resident CD4 T_RM_ might reveal useful to provide local help to CD8 ACT products, and by that their function and survival.

## ACT for Tumor Therapy: Knock-Knock, May We Come In?

**FIGURE 1:**
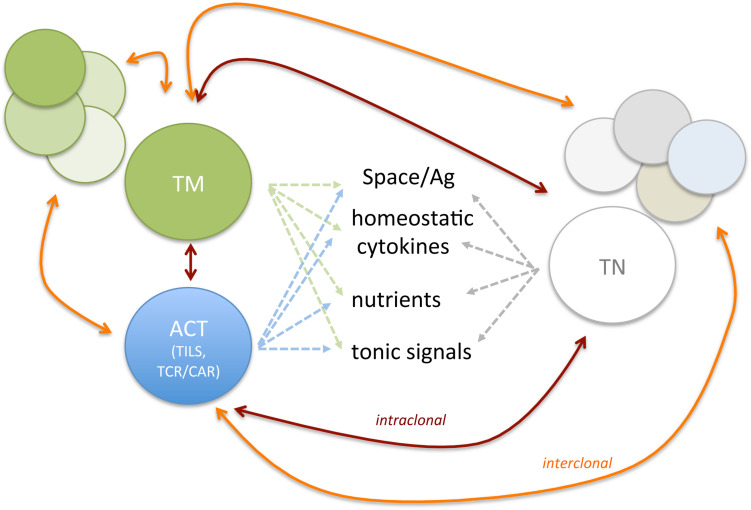
Endogenous and ACT T cell subsets compete for space and resources. Are new comers strong enough to outcompete long-term resident? The figure depicts possible competition among T_N_, Ag-experienced T cells (T_CM_, T_EM_, T_EFF_, T_RM_, T_EX_, and T_reg_), and ACT. Both intra-clonal competition (among T cell subsets expressing the same TCR, red lines) and inter-clonal competition (among T cell subsets with different TCR, orange lines) might shape persistence of newly infused T cells and the composition of endogenous T cell repertoire.

**FIGURE 2:**
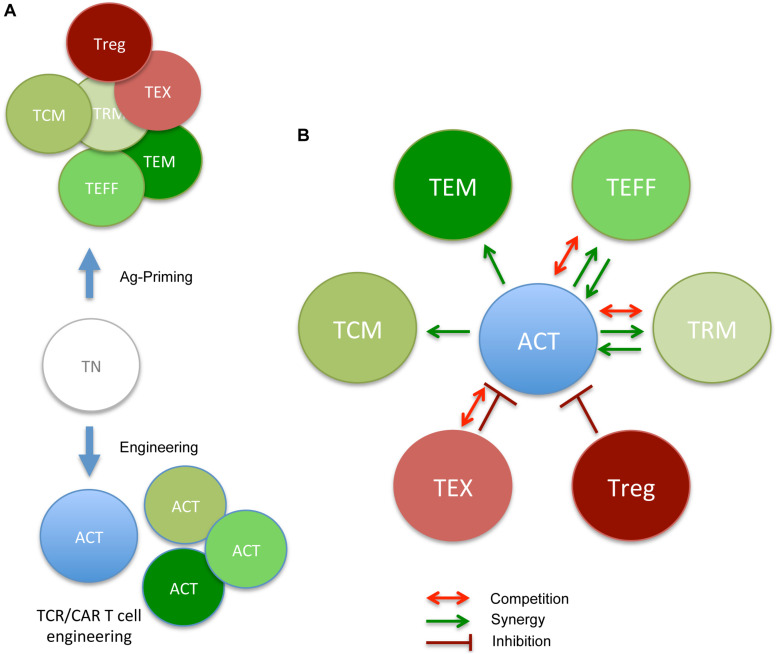
ACT-derived T cell products and cross-talk with pre-existing memory/Ag-experienced subsets (the good, the bad, and the ugly neighbors). **(A)** During *in vivo* Ag priming or *in vitro* engineering/expansion, T_N_ acquire diverse phenotypes, reflecting various degrees of differentiation (T_CM_, T_EM_, T_EFF_, T_RM_, T_EX_, and T_reg_). Each subset reveals defined requirements for proper effector function and survival. **(B)** In the context of ACT, newly infused T cells will interact with pre-existing subsets, and either benefit from good memory subsets or take advantage of them (T_CM_, T_EM_, T_EFF_, and T_RM_) in promoting therapeutic anti-tumor responses (green arrows) or be inhibited by nasty ones (T_EX_ and T_reg_). In some cases, competing for nutrients/survival signal (Figure 1) might also dim ACT efficacy (red arrows). Putative collaborative and antagonistic relationship between pre-existing memory subsets and ACT cell products are depicted.

Adoptive T cell therapy for tumor therapy is generally provided in the context of allogeneic or autologous settings. In the case of HLA-matched allogeneic donors, mature T cells comprise undefined T_N_ and T_M_ populations transferred at the time of stem cell transplant or shortly after (81–83). According to their nature and T_M_ composition, allogeneic T cells can provide graft-versus-tumor effects and also graft-versus-host disease owing to the presence of T cell reactive to minor histocompatibility antigens (84). In the autologous settings, instead, T cell products can be generated by the expansion of tumor-reactive cells isolated from tumor specimens (TILs) or by the genetic engineering of peripheral blood T cells with TCR or CAR, an antibody-derived single-chain variable fragment fused to T cell signaling domain(s), specific for tumor-specific/associated Ags (46, 85, 86). The phenotype of ACT T cell products can impact on therapy efficacy (87). Given that natural and manufactured T_M_ share similar requirements, they might compete for space, nutrients, cytokines, or TCR-engaging ligands *in vivo* (Figure 1). According to manufacturing culture conditions, T cells can acquire various T_M_ features. Which T_M_ cell subset represents the most effective in driving durable cures in cancer patients has been debated and remains to be fully elucidated and might vary according to the cancer type/state. Preclinical and clinical studies have shown that less differentiated T_SCM_ and T_CM_ display better expansion, persistence, and antitumor activity *in vivo* when compared with fully differentiated T_EFF_ (88–90). Accordingly, in retrospective analysis of ACT trials, more favorable objective clinical responses were found with less differentiated T cell products (88, 91). Although initial studies adopted IL-2 to support the *in vitro* expansion of engineered T cells, it soon became evident that T cell products had limited survival potential when transferred *in vivo* (92). Rather, shorter expansion times, and the use of IL-7, IL-15, and IL-21 provided T cells with longer persistence *in vivo* (92). As T_SCM_ and T_CM_ are found in limited number in the peripheral blood and at the tumor site, *in vitro* methods have been defined to generate them starting from T_N_ precursors. These include polyclonal activation (αCD3/28 antibody−conjugated beads), homeostatic cytokines (IL-7, IL-15, and IL-21), (93–95) inhibitors of specific signaling pathways (such as GSK3β, AKT, or mTOR) (96, 97) or epigenetic regulators, (98) and nutrients/metabolites aimed at arresting terminal differentiation and promoting memory stem cell phenotype, during *ex vivo* T cell expansion. The same extracellular cues that guide the manufacturing tumor-reactive lymphocyte continue to affect the activity of adoptively transferred T cells, which, as introduced in the previous section, compete with pre-existing T_M_ subsets and the tumor microenvironment (TME), once re-infused in patients. We argue that this competition might have direct consequences on *in vivo* differentiation and survival of ACT products and their therapeutic effect (Figure 2).

## Cell Intrinsic and Cell Extrinsic Events With the Potential to Shape the Host–ACT T_M_ Cross-Talk

Both cell-intrinsic and cell-extrinsic events shape T_M_ differentiation, survival, and functions. In this paragraph, we review some of the available evidence that support the impact of clonal abundance, TCR affinity, and availability of cytokines and nutrients on T_M_ behavior and their possible implication in the host–ACT T_M_ cross-talk. As TILs and TCR-engineered T cells recognize MHC restricted antigens, while CAR-T cells bind surface antigens via MHC-independent mechanisms, the impact of such critical determinants might vary and be relative to each given ACT product.

### Clonal Abundance

In natural immunity, initial clonal abundance, i.e., the frequency at which any given TCR is represented within the polyclonal repertoire, and the relative TCR affinity for cognate antigens have been shown to give rise to intraclonal and interclonal competition and shape the T_M_ repertoire [recently discussed in (99)]. Data have shown that natural differences in the size of foreign Ag-specific T cell populations have direct consequences on the magnitude of the effector and memory response (100, 101). Intraclonal competition has been shown to affect the expansion and accumulation of both T_N_ and T_M_ naïve cells and to block the proliferation of adoptively transferred CD8 T cells (102). In cases where T cells exceed the relatively narrow physiologic range, intraclonal competition was observed with consequences on overall T_M_ survival and expansion potential (103). Among several factors, competition for Ag and/or access to Ag-bearing APC played a role, (104–106) which caused suboptimal T cell expansion, and defective generation of effector and memory T cells against foreign pathogen and tumors (107–109). In the case of CD8 T cells specific for a tumor-associated self-Ag, increasing naïve T cell frequency at supra-physiological precursor frequencies was reported to ameliorate responses to tumor-specific vaccination and therapeutic effects. Nevertheless, above a certain threshold, intraclonal competition was observed, and this limited protective immunity (110). We also found, in a model of spontaneous prostate cancer development, that increasing the number of TCR-transduced T cells or repeating their administration over time did not significantly increase the relative abundance nor their therapeutic potential (111). Of note, in some models, sequential administration of tumor-specific CTL proved more efficacious than the single initial injection of an equivalent CTL number (112). Thus, data support the notion that intraclonal competition regulates both naturally occurring and ACT-derived antigen-specific CD8 T_M_.

Intraclonal competition was also reported to regulate relative abundance of T_N_ and T_M_ CD4 T cells (and in the case of tumor/self Ag-specific naïve CD4 T cells). By transferring different numbers of tumor-associated self-Ag-specific TCR transgenic CD4 T cells, Malandro and co-authors showed that initial precursor frequency inversely correlated with *in vivo* expansion and functional outcomes. Although at low precursor frequency CD4 T cells specific for a tumor-associated self-Ag underwent robust proliferation, they acquired an irreversible exhausted phenotype (113). In contrast, at higher precursor frequencies, T cells showed a poor expansion potential, but preserved an optimal cytokine-secretion profile and antitumor activity. Interestingly, the authors showed that above a certain threshold, intraclonal CD4–CD4 T cell help cooperation became evident (113). It is tempting to speculate that increasing the frequency of tumor-specific CD4 T helper cells might provide a selective advantage to both naturally occurring and ACT-derived T_M_. Whether increasing the availability of CD4 T cell help might be beneficial to ACT approaches, and whether predefined CD4 and/or CD8 T cell compositions at various degree of differentiation would ameliorate ACT therapeutic potential is currently being investigated in a number of ongoing trials (91, 114).

### TCR Affinity/Avidity

Availability of the cognate antigen or access to cross-reactive self-Ag appears to be a critical determinant in dictating clonal abundance within a memory subset and/or the emergence of high/low affinity TCRs. In the case of CD4 T cells, weak TCR signals from self-peptide MHCII ligands are important for T_M_ survival, and this becomes limiting in the presence of high frequencies of CD4 T cells specific for the same ligand (103, 115). In the case of CD8 cells, this is critical for shaping the T_M_ repertoire, but less so for T_M_ maintenance, which predominantly depends on cytokines such as IL-15 and IL-7 (116, 117). Nevertheless, TCR affinity/avidity contributes to clonal selection. It regulates access to vital co-stimulatory molecules, cytokines, and nutrients, (99) and it determines that clonotypes with higher affinity (and slower TCR–ligand dissociation rates) acutely increase in frequencies within T_M_ pools, above levels reached during the course of anti-viral immunity starting from naïve precursors (118, 119). During acute murine cytomegalovirus (CMV) infection, for instance, a subset of high-avidity virus-specific CD8 T cells typically increases in size (clonal dominance) and simultaneously establishes a large pool of effector memory T cells able to outcompete lower avidity CD8 T cells (120, 121). This mechanism has now been defined as “memory inflation” and documented over the course of several viral infections (122, 123). In a recent study, Schober and colleagues studied TCR repertoire evolution in the context of latent CMV infection and found that, although high-affinity TCRs dominated T cell responses at early times after infection, low affinity TCRs emerged over time, owing to cellular differentiation and senescence (and not exhaustion) of high affinity ones (124). In a recent publication, Poschke and coauthors exploited TCR deep sequencing to characterize TILs before and after *in vitro* culture and found that dominant T cell clones were lost during TIL culture because of poor expansion potential, in favor of less represented ones (125). The authors argue that spatial heterogeneity of the tumor T cell repertoire, as well as differences in intrinsic *in vitro* growth capacity between individual T cell clones, influenced the T cell preparation. In melanoma, the most abundant T cell clones were found to be tumor reactive, and yet, neo-antigen-reactive T cells, of possible highest affinity, were gradually lost during TIL expansion. Whether this occurs also over the course of natural evolution of anti-tumor immunity and/or under the pressure of ACT remains to be determined. Yet, it is tempting to speculate that in some cases a switch in dominance toward low-affinity TCR T cells might indeed take place during the editing process, especially under the pressure of high-affinity ACT T cells and precede and/or account for final tumor escape.

The remodeling of the host T cell repertoire was observed in ACT for both mouse and human CMV. In mice, adoptively transferred T cells were shown to restrict the repertoire of host-derived T cells via competitive mechanisms, supporting clonal dominance of T_M_ ACT cells over endogenous memory cells (126). In the setting of anti-CMV-specific ACT, T_M_ influenced further responses by endogenous CMV-specific T cells in organ transplant recipients (127). This was best observed in patients who had an unbiased TCR repertoire before the transplant, and correlated with therapy efficacy, likely owing to further expansion of viral-specific T cells. Instead, non-responding recipients revealed a pre-transplant biased peripheral T cell repertoire, which was not influenced by ACT. This indicates the ability of ACT to promote a restructuring of the T cell pool, given proper immune cell representation pre-ACT. Because adoptively transferred T cells synergize with endogenous responses (128) (discussed in following paragraph), future studies would be needed to understand how TCR/CAR-T cell impact on the representation of pre-existing and newly generated tumor-reactive T_M_ cells.

Thus, when considering ACT, a detailed characterization of host immune competence might help predict efficacy, and also instruct optimal T cell product composition. Of note, CAR-T cells might be expected to be less sensitive to the competition with endogenous T_M_ subsets than TILs or TCR redirected T cells, at least for TCR engaging ligands and deriving tonic stimulation. In the case of CAR-T cells, antigen-independent tonic signaling has been shown to result from spontaneous clustering of CAR molecules. However, in contrast to tonic TCR signaling, this event was associated with augmented T cell apoptosis, exhaustion, and impaired antitumor effects (129, 130). As preclinical studies suggest that CAR–TCR interactions are a prerequisite for optimal CAR-driven T cell activation, (131) it remains possible that survival of CAR-T_M_ cells is controlled by the same signals that support TCR T cells. If this would be the case, then also CAR-T cell might be sensitive to surrounding endogenous T_M_ subsets.

### Cytokines

In the context of natural immunity, competition for γ_c_ cytokines, principally IL-7 and IL-15, regulates the balance of CD4 and CD8 T_N_ and T_M_ cells, homeostatic proliferation, and survival (132–134). Only those T cells receiving sufficient signaling escape the apoptotic process and proliferate (135–137). IL-7 mostly supports CD4 and CD8 T_M_, whereas IL-15 also promotes their homeostatic proliferation. Given the higher expression of IL-2Rβ, CD8 T_M_ cells are more sensitive to IL-15, and outcompete CD4 T_M_ and T_N_ cell subsets during acute infections and homeostatic proliferation (138, 139).

T cells transferred in the setting of ACT are also sensitive to γ_c_-cytokine availability. Accordingly, cytokines like IL-2, IL-7, IL-15, and IL-21 are fundamental both for generation of the ACT cell products and to increase the efficacy and the duration of the anti-tumor response *in vivo*. Administration of low-dose IL-2 to the patient after ACT therapy has generally been used to enhance the *in vivo* persistence of the newly adoptively transferred T cells which has been shown to translate in a favorable clinical outcome (140). Although initial studies adopted IL-2 to support the *in vitro* expansion of engineered T cells, it soon became evident that T cell product had limited survival when transferred *in vivo* (92). Rather, shorter expansion times and the use of IL-7 and IL-15 mediated the selective expansion of CD4 and CD8 T cells, while limiting the representation of T_reg_ cells, resulting in longer persistence *in vivo* (141–143). Because of their capacity to support, both *in vivo* and *ex vivo*, T_M_ cell generation and homeostatic proliferation, IL-7 and IL-15 are now being evaluated in human clinical trials. IL-21 also plays an important role in ACT manufacturing based on its ability to significantly enhance the *ex vivo* generation and TCR affinity of T_M_ cells (144, 145).

As in the case of TCR engaging ligands, endogenous and ACT T_M_ might compete for cytokine availability. Thus, once more the T cell repertoire before ACT might influence ACT efficacy, and vice versa recently infused T cells could impact on endogenous subsets. Accordingly, lymphodepletion by various pre-conditioning protocols has been shown to promote both the engraftment and the long-term persistence of TILs, TCR, (142, 146, 147) and also of CAR-T cells (148–150) by lowering the number of immune cell subsets, which compete with transferred tumor-reactive T_M_ for the γ_c_-cytokine binding (142). Clinical trials in melanoma patients first indicated that TIL persistence was improved by preconditioning of the patients with lymphodepleting strategies based on total body irradiation (151, 152). Preclinical models further demonstrated that lymphodepleting regimens improve engraftment and functionality of transferred T cells prolonging their survival, via IL-15-dependent signaling (153). The competition for homeostatic cytokines might also evolve in a “metabolic competition” that might render ACT T_M_ dysfunctional and metabolically exhausted. Indeed, IL-15 also regulates T_M_ metabolic stability (154). In this respect, data indicate that IL-7 administration following cyclophosphamide preconditioning supports the expansion of polyfunctional tumor-specific CD4 T cells (155). Thus, in this scenario, we speculate that lymphodepletion is able to contribute to effective anti-tumor immunity because, in addition to be critical to eliminate immune-suppressive cells (i.e., MDSCs, TAM, T_EX_, and T_regs_) and decrease the metabolic competition in the TME for IL-7, IL-15, and nutrients, it bears the potential to support both ACT T_M_ and possibly also endogenous T_N_ and T_M_.

### Nutrients

Nutrient availability is a requisite for proper T_M_ function and long-term persistence. T_M_ cells own a unique metabolic signature. On Ag encounter, T cells engage OXPHOS, glutaminolysis, and glycolysis to fulfill bioenergetic and biosynthetic demands needed to support proliferation and effector functions. After Ag clearance, T_EFF_ cells reduce their metabolic demands and dependence on glycolysis, and gradually reset back from an anabolic to a catabolic state, typical of long-lived T_M_ cells. At difference with T_EFF_ cells, T_CM_ cells are mostly quiescent and adopt a metabolic profile similar to T_N_ cells, whereby they rely on mitochondrial metabolism and fatty acid oxidation to support their persistence and functions (156, 157). T_M_ cells also possess greater mitochondrial mass and enhanced Spare Respiratory Capacity, (158) two key metabolic features important not only for their development and long-term survival but also for their rapid recall ability.

In the context of ACT, metabolic fitness plays an important role. T cells with high metabolic activity and glycolytic rate are endowed with potent T_EFF_ functions (i.e., capable of cytokine production and rapid proliferation). The identification of the metabolic pathways critical to T_M_ survival and functions supported the concept that *in vitro* metabolic reprogramming could impact *in vivo* tumor activity. As a consequence, several approaches targeting T cell metabolism *in vitro* and *in vivo* by targeted delivery of metabolism-modulating compounds to the TME have been investigated for effective cancer immunotherapy and recently reviewed (93, 94).

Nevertheless, within the TME, tumor cells and/or other infiltrating immune subsets share metabolic requirements, which are overlapping with those of ACT products. Tumor cells indeed frequently share a similar glycolytic metabolic profile with activated T cells, and compete with them for fundamental nutrients, hindering the ability of effector T cells to meet energetic needs. Two studies showed that highly glycolytic tumors can deplete glucose levels in the TME, dampening the ability of T cells to maintain anabolic growth signaling and produce inflammatory cytokines (159, 160). Moreover, by consuming glucose and producing cAMP, tumors limit T_EFF_ functions and instead promote T cell senescence (161). Additional studies highlight that high rates of amino acid uptake by tumor cells could potentially inhibit the anti-tumor T cell response because amino acids are crucial nutrients that support T cell proliferation and effector functions (162, 163). There is also emerging evidence that some tumors uptake fatty acids with high rates to maintain their proliferation (164). Given that fatty acids have a central role in T cell memory differentiation and function, (165) lipids may be another nutrient that T cells must compete for in the TME.

In the context of this competition, it is worth noticing that both CD8 T_RM_ and T_CM_ cells depend on mitochondrial FAO and OXPHOS. However, to fuel this metabolic pathway, CD8 T_RM_ cells strictly depend on uptake of exogenous fatty acids, (166) whereas CD8 T_CM_ cells rather use extracellular glucose to fuel this process. Thus, CD8 T_RM_ cells might be engaged in a metabolic competition with adoptively transferred CD8 T_CM_ cells to fuel mitochondrial metabolism. Whether such competition for nutrient uptake impacts on ACT product functionality remains to be investigated.

The type of nutrients available to T cells, such as glucose, lipids, and amino acid, influences the differentiation program, functional properties, and shape their ability to control tumor progression (167). Although T cell metabolic reprogramming to a given metabolic fitness might be achieved during manufacturing, it should be considered that programming T cells *in vitro* might have a detrimental outcome *in vivo*, as the tumor itself, stromal cells, and/or other infiltrating immune cells compete for critical nutrients. For instance, T cells addicted to glycolysis during *in vitro* culture might experience nutrient deprivation when transplanted in the host and die because of insufficient glucose availability. Therefore, to mount an effective anti-tumor response, ACT products should retain the metabolic flexibility to adjust to nutrient availability. In this scenario, insights into the metabolic characterization of the TME might help inform *in vitro* metabolic re-programming of tumor-specific T cells to ameliorate their persistence and long-term survival in a metabolic unfavorable TME.

### To Remember or To Forget: Good, Bad, and Ugly Memories

Pre-clinical and clinical studies have shown that pre-existing memory T cells contribute to the efficacy of immunotherapy and radio-immunotherapy (168–171). Several evidences indicate that also in the case of ACT with TCR/CAR engineered products, host T cells contribute to the therapeutic anti-tumor responses. The heterogeneity within the responding pool of T_M_, owing to the ability of the cells to integrate several signals (TCR, co-stimulatory molecules, and cytokines), their differentiation status, and their relative fitness in an environment of rapidly expanding cells competing for the same resources, is thus likely to impact on the final therapeutic outcome.

Could then pre-existing memory or on a more general definition, Ag-experienced T cell subsets be stratified according to their impact on ACT efficacy? We speculate that this could be the case, in relation to their relative function in protective responses, as they could improve or hinder ACT products. “Good memories” might be represented by those subsets of cells, endowed with effector capabilities and able to synergize with tumor-specific T cells provided by ACT. For instance, pre-existing T_M_ or T_RM_ might respond to pro-inflammatory signals generated in response to intra-tumoral activation of TCR/CAR engineered T cells, (172) and contribute to anti-tumor immunity by conditioning the immune milieu and/or exerting direct anti-tumor activity. In a recent report, Walsh et al. showed that adoptively transferred T cells synergized with endogenous T cells, which were instrumental to prevent immune escape of Ag-loss variants. Post-transplant tumor-specific vaccination supported better tumor infiltration and prolonged survival of tumor-reactive lymphocytes, likely promoting intra-tumoral responses and Ag cross-presentation (128). Local activation of T_RM_ cells might in turn favor the spreading of circulating CTL responses against tumor-derived neo- and self-antigens (173). We also found that tumor-specific vaccination promoted optimal anti-tumor immunity when applied after allogeneic hematopoietic cell transplantation or ACT with tumor-redirected TCR engineered T cells. Also in these settings, in addition to engineered T cells, non-transduced cell subsets were revealed capable of IFN-γ and Granzyme-B expression (174–177). Similarly, Ma and co-authors found that the implementation of a vaccine boosting via the CAR (using a smart by-specific vaccinable CAR) enhanced CAR T cell expansion and also favored the recruitment of additional specificities (178). It is possible that infiltrating T cells, positively selected in the thymus by virtue of expressing TCRs with a low/intermediate affinity for self-Ags, are indeed able to recognize such antigens on tumor cells, or that TCR/CAR-induced inflammatory environment promotes effector function by bystander cells via non-Ag-specific mechanisms (179–181). In this respect, it is worth mentioning that robust and iterative stimulation of memory self-specific CD8 T cells reverted tolerance to self in the context of acute infection, and promoted anti-tumor immunity, without precipitating autoimmune manifestations (182). This is reminiscent of the synergy between T cells specific for a tumor and a Y chromosome–derived self-antigen in the context of allogeneic hematopoietic cell transplantation and in ACT with TCR-redirected T cells (174, 175). These studies suggest to exploit the use of pre-existing or newly infused T_M_ possibly reactive to self/tumor-associated Ags in ACT of tumors.

Viral-specific memory T cells could also come to help. It is known that both mouse and human tumors are commonly surveyed by memory T cells specific for previously encountered viral infections (183). A recently published manuscript showed that these functional T cells can be specifically reactivated via the local delivery of viral peptides, which caused a local inflammatory environment capable of activating both the innate and adaptive immunity, leading to tumor growth arrest. Immunization with viral peptides sensitized mice to PD-L1 checkpoint blockade promoting the elimination of otherwise resistant tumors (169). Thus, viral-specific memory T cells, if appropriately activated, might synergize with ACT.

Finally, resident T_RM_, bystander memory subsets, and virtual memory T cells might also play a role, as capable of responding to pro-inflammatory cytokines, known to lower the threshold for T cell activation and/or induce TCR-independent effector functions (180, 184). Supporting this, CAR T cells engineered to express IL-12 and/or IL-18 (TRUCK T cells) have proven more effective than those only expressing the tumor-specific CAR (185, 186). TCR could cross-recognize multiple Ags or respond to unrelated Ags, self-Ag, or environmental Ags (187, 188). Virtual memory T cells can produce IFN-γ and are capable of Ag-independent lytic activity, in response to the inflammatory milieu alone, i.e., when stimulated with IL-12, IL-15, and IL-18 (189).

**FIGURE 3:**
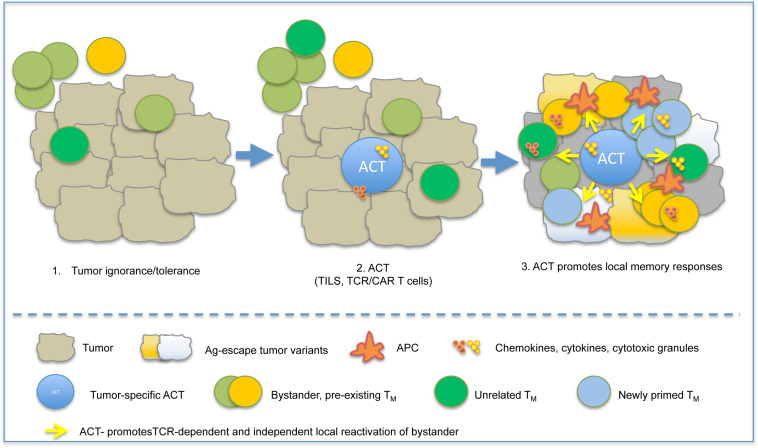
ACT instructs protective immune responses in tumors. A schematic model depicting the possible influence of ACT on pre-existing memory is depicted. Shortly after infusion, ACT-derived T_EFF_ recognize tumor antigen, cause tumor cell death and release of pro-inflammatory cytokines (IFN-γ, TNF-α), and promote local inflammation. This causes recruitment of APC and bystander memory subsets, and their local reactivation by either TCR-dependent or TCR-independent mechanisms.

Although in several instances the cellular and molecular mechanisms at the base of the synergistic effect need further investigation, bystander T memory cells were frequently found within the tumor infiltrates, (180) suggesting that pre-existing memory cells, unrelated to the cognate tumor-associated Ag targeted by TILs or TCR/CAR T cells, might contribute to anti-tumor immunity in ACT settings (Figure 3).

Along this line, it should be mentioned that unfortunately, also “bad memories” exist, which may impair the development of new memories. This was shown to be the case in the well-recognized phenomenon of “original antigenic sin,” where an existing immune response prevents the initiation of a later, cross-reactive but independent immune response. This phenomenon, which was described for antiviral CD8 T cell immune responses (190, 191) and in vaccinated mice, owing to the ability of pre-existing effector cells to eliminate Ag-bearing dendritic cells, might play a role in limiting propagation of anti-tumor protective memory responses. Accordingly, we found that anti-tumor CD4 T cell responses limited efficacy of active vaccination in tumor-bearing mice, (192) supporting the possibility that in some cases pre-existing memory might counteract a newly born one.

Although in real life it is well conceivable that these “bad memories” might protect individuals from recurrent infection/disease, in the context of tumor immunity, they might represent an obstacle to therapeutic efficacy.

Finally, even if anergic and T_EX_ cells, putative representative of bad memory T cell subsets, might appear to have no apparent function, they might consume useful resources needed for the efficacy of ACT cell products. This has been shown for T_reg_ subsets, which, for instance, can induce T_EFF_ cell senescence by competing for glucose and inducing DNA damage (193). Hence, this subset might prove to be an “ugly neighbor,” capable of providing active suppression and inhibiting tumor recognition by ACT T cell products right from the start.

## Conclusion

Given the aforementioned evidence, should pre-existent memory be considered before adoptive T therapy? The authors believe that it should.

The degree of heterogeneity within a T cell pool depends on the integration of signals from the TCR, co-stimulatory molecules, cytokines and nutrients, and also on the relative cell fitness within an environment competing for the same resources. Newly infused T cells would need to face and adapt to such pre-existing conditions, to engraft and exert proper anti-tumor activity. The concept of immunological memory foresees that pre-existing memory T cells would be beneficial for protection against reinfection with the same pathogens, because Ag-specific memory T cells would be numerically increased when compared to endogenous ones, have widened anatomical distribution, and respond more quickly, conferring rapid clearance of the infectious agent. Nevertheless, previous encounters with the Ag has the potential to generate in addition to good memories (T_CM_, T_EFF_, and T_RM_), also bad and ugly memories (T_EX_, T_reg_, and in some instances tumor-associated T_RM_), with opposite effects on recall responses, and on the efficacy of adoptive T cell therapy. The quality and quantity of adoptively transferred cells are also important parameters to consider when optimizing such a treatment. “*The more is the better*” might not be the right choice, as cells should find sufficient space and support (TCR/CAR engaging ligands, homeostatic cytokines, nutrients) in spite of host–cell competition. Modeling tumor–immune system competition might help predict responses *in vivo* (112, 194). Would also provoking immunological amnesia promote therapeutic efficacy of T cell products in the context of ACT? We speculate this might not be entirely the case because as in real life, retaining positive memories might help. The finding that endogenous T cells cooperate with TCR/CAR-redirected T cells supports this statement (128, 195). Rather, strategies suitable to evoke selective amnesia from *bad* memories (i.e., capable of depleting/inhibiting regulatory subsets, and/or overcome competition for space or nutrients) would empower *good* ones and amplify therapeutic effects of current ACT products. As an alternative, synthetic biology and genetic engineering might help design T cell products insensitive to competition or able to metabolically adapt to hostile TME. Likewise, understanding and exploiting CD4 and CD8 T_M_ representation, both within the ACT product, and the pre-existing endogenous repertoire, might open new avenues of intervention. Thus, interesting challenges ahead will be to understand the cross-talk and homeostatic regulation between adoptively transferred T cells and endogenous ones to define strategies to eliminate any unneeded immunological bad memories, and take advantage of available local resources. This will foster productive synergies and supportive environments to render ACT products highly functional.

## Author Contributions

All authors listed have made a substantial, direct and intellectual contribution to the work, and approved it for publication.

## Conflict of Interest

The authors declare that the research was conducted in the absence of any commercial or financial relationships that could be construed as a potential conflict of interest. The handling editor declared a past co-authorship with one of the authors AM.
